# The Civil Hospitals and the War Office

**Published:** 1916-04-29

**Authors:** 


### The Hospital

The Workers' Newspaper of Administrative Medicine and Institutional Life, Administration, National Insurance and Health.

No. 1559, Vol. LX. SATURDAY, APRIL 29, 1916. PRICE ONE PENNY.

### THE CIVIL HOSPITALS AND THE WAR OFFICE.

The critic is just now very much abroad in the land and his voice is heard in all the newspapers. To do him full justice, he is not wanting in constructive aim, for he usually has a scheme, or even half a dozen, up his sleeve. The slowness and the stupidity of the official world is his favourite text, and if only his advice were followed difficulties would disappear as by magic. One is sometimes driven to wonder how so much diplomatic, financial, political, naval, and military ability can be collected in a single newspaper office. No members of the community are better equipped by training and temperament than are medical practitioners to discount hasty and ill-informed judgments and to recognise the difficulties of experts. Much of their own work is beyond the reach of mere popular appreciation, and the difficulties of many of the situations they have to meet cannot be explained to the amateur. Hence the atmosphere of medicine tends to breed patience and sympathy towards those who have to carry a heavy burden of responsibility and who, though exposed to public criticism, are prevented from a full attempt to explain their decisions. We trust that in these directions we do not fall below the level of our colleagues, and certainly in any criticisms we have passed on any of the official arrangements our object has been not to hinder but to help. This claim applies in particular to what has appeared previously in these columns relative to the reception of sick and wounded soldiers in the civil hospitals. From the outset we have allowed that the circumstances which existed in the early days of the war justified the appeal which the War Office made to the hospitals; and, similarly, we have recognised that the hospitals were right in responding to this appeal. Sick and wounded soldiers were of the first interest to the nation, and it was only just that they should receive hospital attention, even though this involved some prejudice to the civil population. As an adequate supply of military hospitals was not then organised it followed inevitably that the soldiers must be received in the civil institutions. The strain on the military medical service was both great and unexpected, and time was needed for the developments necessary to meet the situation.

But what was seemly and, indeed, inevitable at the commencement of the war, assumes another character at the present date. There has been adequate time for the organisation of military hospitals, and hence on this ground alone it might have been anticipated that the civil hospitals would now be free to pursue their proper work. Unhappily, experience shows that this is not the case. Both in London and in the country soldiers in considerable numbers are being sent into the ordinary hospital wards, with the necessary consequence that a corresponding proportion of patients from the poorer classes have to be denied admission. Clearly such a state of matters after twenty months of war needs justification. But this is not all. Not only has there been time for the organisation of military hospitals, but military hospitals have, as a matter of fact, been organised and are in existence. Yet though they are in existence many of their beds are unoccupied and some of their wards are closed. At this moment some of these hospitals are not half full, and this state of matters has existed during the whole of the past winter. Surely in these circumstances it is little short of an outrage that the rights and privileges of the sick poor should be unnecessarily invaded. We need not say that we grudge no comfort and no skill given to our soldiers. But from what has already been said here it is obvious that there is abundance of provision for the soldiers, and that only when this is exhausted can the use of the civil hospitals for military purposes be justified. It may appear ungracious to write in this tone, but we have in these columns a special duty to the hospitals and to those for whom they are supported, and it is in discharge of this duty that plain speech is incumbent on us. The short word is that the hospitals are at this moment and to a large extent being taken away from those for whose help they were intended, and they are being taken away unnecessarily seeing that the War Office has in the military hospitals numbers of unoccupied beds at its disposal.

There is still another word to be said. It is that a considerable proportion of the military patients at present sent into the civil hospitals are by no means seriously ill. Whole wards may be seen in which there is not a single patient confined to bed. The sound of music and dancing is to be heard, or a crowd of men are enjoying their tobacco seated around a comfortable fire; or perchance the ward is for the moment empty as the patients have all gone to the theatre. In other words, many of these men want rest, good food, fresh air and sunshine, and a mere minimum of medical or surgical treatment. In ordinary circumstances they would not be deemed suitable for in-patient care. By all means let them be provided with everything which will promote their welfare and which will witness to them the country's gratitude. But to obtain these ends it is not necessary to sacrifice the interests of the sick-poor. Yet this is exactly what is being done under the pressure of the War Office. We cannot think that the governing bodies of the hospitals can altogether escape responsibility. Their position is indeed a difficult one, and to refuse anything to our soldiers has an ungracious quality. But the governors are charged with a trust, and it is theirs to see that the interests placed in their hands are fully preserved. No benevolence of motive and no patriotic glow can excuse a departure from this position. Since it is known that military hospitals are not being anything like fully utilised, the time has come for the governors of the civil hospitals to stand to their duty and to intimate to the War Office that what was granted under the pressure of an emergency cannot be continued without protest and criticism merely to suit the official convenience.

